# Acquisition and Loss of CTX-M-Producing and Non-Producing Escherichia coli in the Fecal Microbiome of Travelers to South Asia

**DOI:** 10.1128/mBio.02408-18

**Published:** 2018-12-11

**Authors:** Edward R. Bevan, Alan McNally, Christopher M. Thomas, Laura J. V. Piddock, Peter M. Hawkey

**Affiliations:** a Institute of Microbiology and Infection, University of Birmingham, Birmingham, United Kingdom; b Public Health England, University Hospitals Birmingham NHS Foundation Trust, Public Health Laboratory, Birmingham, United Kingdom; Lahey Hospital and Medical Center

**Keywords:** *Escherichia coli*, colonization, microbiome, travel

## Abstract

Over 80% of travelers from the United Kingdom to the Indian subcontinent acquire CTX-M-producing Escherichia coli (CTX-M-EC), but the mechanism of CTX-M-EC acquisition is poorly understood. We aimed to investigate the dynamics of CTX-M-EC acquisition in healthy travelers and how this relates to populations of non-CTX-M-EC in the fecal microbiome. This is a prospective observational study of healthy volunteers traveling from the United Kingdom to South Asia. Fecal samples were collected pre- and post-travel at several time points up to 12 months post-travel. A toothpicking experiment was used to determine the proportion of cephalosporin-sensitive E. coli in fecal samples containing CTX-M-EC. MLST and SNP type of pre-travel and post-travel E. coli were deduced by WGS. CTX-M-EC was acquired by 89% (16/18) of volunteers. Polyclonal acquisition of CTX-M-EC was seen in 8/15 volunteers (all had >3 STs across post-travel samples), suggesting multiple acquisition events. Indistinguishable CTX-M-EC clones (zero SNPs apart) are detectable in serial fecal samples up to 7 months after travel, indicating stable maintenance in the fecal microbiome on return to the United Kingdom in the absence of selective pressure. CTX-M-EC-containing samples were often co-colonized with novel, non-CTX-M strains after travel, indicating that acquisition of non-CTX-M-EC occurs alongside CTX-M-EC. The same pre-travel non-CTX-M strains (<10 SNPs apart) were found in post-travel fecal samples after CTX-M-EC had been lost, suggesting return of the fecal microbiome to the pre-travel state and long-term persistence of minority strains in travelers who acquire CTX-M-EC.

## Abstract (precis)

Escherichia coli strains which produce CTX-M extended-spectrum beta-lactamases are endemic as colonizers of humans and in the environment in South Asia. This study demonstrates that acquisition of CTX-M-producing E. coli (CTX-M-EC) in travelers from the United Kingdom to South Asia is polyclonal, which is likely due to multiple acquisition events from contaminated food and drinking water during travel. CTX-M-EC frequently persists in the fecal microbiome for at least 1 year after acquisition, often alongside newly acquired non-CTX-M E. coli strains. In travelers who acquire CTX-M-EC, pre-travel non-CTX-M E. coli remains as a minority population in the gut until the CTX-M-EC strains are lost. The non-CTX-M strains are then reestablished as the predominant E. coli population. This study has shed light on the dynamics of CTX-M-EC acquisition, colonization, and loss after travel. Future work involving manipulation of nonvirulent resident E. coli could be used to prevent colonization with antibiotic-resistant E. coli.

## INTRODUCTION

The global prevalence of CTX-M-producing Escherichia coli (CTX-M-EC) is rising, thereby increasing the use of carbapenem antibiotics and acting as a driver for the emergence of carbapenemase-producing *Enterobacteriaceae* (CPE) (1). Anthropogenic factors such as low levels of sanitation, poor standards of animal husbandry, and the use of antibiotics in humans and animals all contribute to the high prevalence of CTX-M-EC in low and middle income countries (2). In the United Kingdom, the prevalence of fecal carriage of CTX-M-producing *Enterobacteriaceae* remains low at 7.3% (3).

International travel plays a key role in the spread of antibiotic-resistant bacteria carried in the human gut (4), and the acquisition of CTX-M-EC by travelers who visit countries with high levels of CTX-M producers in the environment is a well-recognized phenomenon (5, 6). In particular, travel to the WHO Southeast Asia region, which includes densely populated countries such as India and Bangladesh, presents the highest risk for acquisition of CTX-M-producing *Enterobacteriaceae* (6). Although acquisition rates by world region, and risk factors for acquisition, have been investigated previously, the dynamics of colonization by ESBL-producing E. coli and non-ESBL-producing E. coli have not.

We undertook a prospective observational cohort study to explore the dynamics of acquisition of CTX-M-EC in travelers from the United Kingdom to South Asia. We used whole-genome sequencing (WGS) to characterize strains before and after travel, providing a novel insight into the changes in populations of E. coli, including the role of non-CTX-M cephalosporin-sensitive E. coli in the human fecal microbiome of returning travelers.

## RESULTS AND DISCUSSION

### Travelers to South Asia have a high CTX-M-EC acquisition rate.

**FIG 1:**
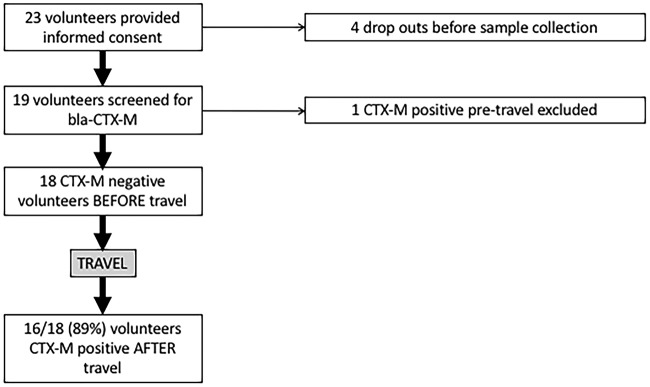
Outline of volunteers recruited and excluded from study with CTX-M-EC acquisition rate post-travel.

Twenty-two volunteers provided informed consent to take part in the study. Three volunteers withdrew from the study before providing samples (Fig. 1). The mean and median ages of volunteers were 34 and 30 years, respectively. After screening volunteer stool samples using selective culture and PCR, 18/19 were *bla*_CTX-M_ negative before travel. The single volunteer found to be carrying CTX-M-15-producing E. coli before travel had been to India in the previous 6 months, which is a known risk factor for colonization in the United Kingdom (3, 7). The primary destinations visited by volunteers were India (68%; 13/19) and Sri Lanka (31%; 6/19). The mean travel duration for all countries was 27 days (median, 21 days). The shortest duration of travel by any volunteer was 10 days, which still resulted in CTX-M-EC acquisition. The majority of volunteers experienced symptoms of gastroenteritis at some point during their trip (68%; 13/19) (Table 1).

**TABLE 1:** Outcomes for 18 volunteers before versus after travel^*a*^

Volunteer	Destination(s)	Pre-travel CTX-M	Post-travel CTX-M	CTX-M genotype	Risk factor(s)	Duration of carriage
1	India	No	Yes	15		1–3 wk
3	India	No	Yes	15		>9 mo*
4	Sri Lanka, Cyprus	No	Yes	14 and 15	G	>5 mo*
5	India	No	Yes	15	G	>12 mo*
6	India	No	Yes	15	G	6 wk
7	India	No	Yes	15	G	2–4 mo
8	India	No	Yes	15	G and Abx	>11 mo*
9	Sri Lanka, India	No	Yes	15	G	>7 mo*
10	Sri Lanka, India	No	No	NA	G	NA
12	India	No	Yes	15	G	2–4 mo
15	India	No	Yes	15	G	>1 wk*
16	India	No	Yes	14 and 15	G	>6 mo*
17	India	No	Yes	15		3–6 wk
18	Sri Lanka, China	No	No	NA	G	NA
19	Sri Lanka, China	No	Yes	14	Abx	>14 days*
20	India	No	Yes	15	G	3–4 mo
21	Pakistan	No	Yes	15	G	1–2 wk
22	Sri Lanka	No	Yes	15		1–3 wk

a Abbreviations and symbols: “No,” lack of CTX-M by culture, PCR, and WGS; “Yes,” *bla*_CTX-M_ detected; G, gastroenteritis symptoms; Abx, antibiotic consumption during travel; *, duration of carriage where a “CTX-M-free” follow-up fecal sample could not be obtained; NA, not applicable.

After travel, *bla*_CTX-M_ had been acquired by 16/18 (89%) volunteers (Fig. 1). Two volunteers acquired E. coli producing both CTX-M-14 and CTX-M-15; one volunteer acquired only CTX-M-14-producing E. coli, while the remaining thirteen acquired only CTX-M-15-producing E. coli (Table 1). Specific *bla*_CTX-M_ genotypes are often strongly associated with specific regions; in particular, CTX-M producers in India are almost entirely comprised of *bla*_CTX-M-15_, whereas in China, *bla*_CTX-M-14_ is the predominant genotype (2). Most volunteers traveled to India; therefore, the high acquisition rate of CTX-M-15-producing E. coli is unsurprising. Volunteers 4 and 19, who acquired *bla*_CTX-M-14_, traveled to China and/or Sri Lanka, where *bla*_CTX-M-14_-producing E. coli strains are common (8, 9). Volunteer 16 also acquired *bla*_CTX-M-14_-producing E. col, but traveled only to India, where *bla*_CTX-M-14_ is a rare genotype; however, this individual traveled widely (Kerala, Karnataka, New Delhi, and Uttarakhand). He also worked in a hospital in Bangalore as a volunteer for 4 weeks, which may have resulted in contact with travelers or residents from areas of the world where CTX-M-14 is common, such as China.

The largest traveler study to date, which investigated 2,001 healthy volunteers (the COMBAT study), found that the country visited with the highest ESBL-EC acquisition rate was India: 89% (70/79) of those who traveled from The Netherlands to India acquired ESBL-producing E. coli (6). Other traveler studies have also shown high acquisition rates of ESBL-producing *Enterobacteriaceae* in those who visit India: prospective studies report acquisition rates of 73% (66/90 participants) (10), 85% (53/62 participants) (11), and 87% (59/68 participants) (12). After WGS and bioinformatic screening of contigs, none of our post-travel isolates carried carbapenemase genes. This is consistent with the COMBAT study, which reported the rate of CPE acquisition in returning travelers as <1% (6).

### CTX-M-EC acquisition is polyclonal.

**FIG 2:**
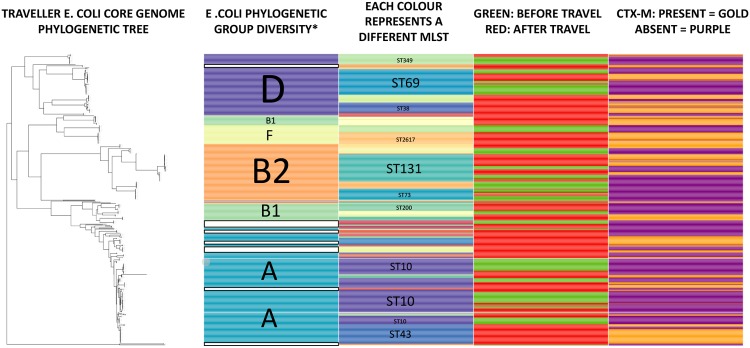
Phylogenetic relationships of pre- and post-travel E. coli strains displaying the phylogenetic groups, majority MLST types, and presence of *bla*_CTX-M_. Four hundred eighty-three E. coli strains from 15 volunteers. *, white bars indicate where phylogenetic group undetermined.

CTX-M-producing E. coli acquired during travel came from a wide range of sequence types (STs) and phylogenetic groups (Fig. 2), in keeping with previous studies (12–16). Among volunteers in our study, we found that 11/15 (73%) acquired CTX-M-EC belonging to phylogenetic group (PG) A. Other common PGs were PG-D (6/15 volunteers, 40%), PG-B2 (5/15, 33%), and PG-B1 (4/15, 27%). ST131 was the majority ST in PG-B2-positive volunteers (4/5 volunteers), with the remaining volunteer carrying ST1193. In terms of E. coli pathogenicity, PG-B2 and PG-D are often extraintestinal pathogenic E. coli (ExPEC), causing urinary sepsis and bacteremia, whereas PG-A and PG-B1 usually lack the pathogenicity genes conferring extraintestinal virulence (17). Our findings are in keeping with previous studies which found a predominance of PG-A in travel-acquired strains (13, 18). Moreover, CTX-M-EC strains belonging to PG-A, PG-D, and PG-B1 were previously found to be commonly acquired after travel (13).

Gut colonization with CTX-M-EC was often polyclonal in those acquiring CTX-M-EC (8/15 volunteers with sequenced E. coli had >1 MLST producing CTX-M [Table 2 and see Table S1 in the supplemental material]). The mean number of acquired E. coli STs per volunteer was 3; 7/15 travelers had >3 E. coli STs with *bla*_CTX-M_. The commonest ST among CTX-M-producing E. coli strains was ST131 (4/15), with other common STs being ST38 (3/15), ST10 (3/15), and ST43 (3/15).

**TABLE 2:**
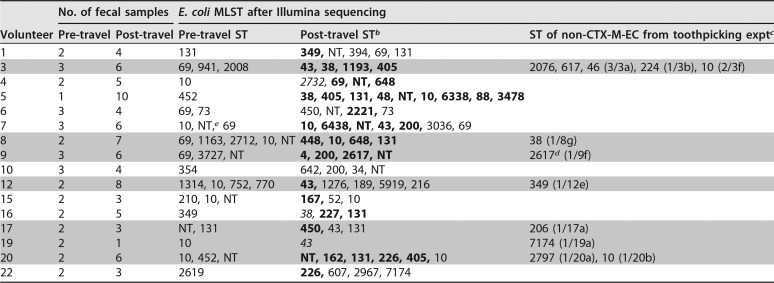
Pre- and post-travel *E. coli* from each volunteer indicating CTX-M-EC, non-CTX-M-EC, and toothpicked strains^*a*^

a Gray shading indicates data from toothpicking experiments.

b Standard font indicates non-CTX-M; boldface indicates CTX-M-15; italic indicates CTX-M-14.

c Text in parentheses, e.g., “(3/3a),” indicates the number of toothpicked colonies which were sequenced, followed by the fecal sample number.

d ST2617, non-CTX-M-EC, in fecal sample 9f, is not the same strain as the CTX-M-producing ST2617, also from volunteer 9 feces (after SNP typing).

e NT, non-typeable.

10.1128/mBio.02408-18.1 TABLE S1 Isolates sequenced pre- and post-travel with corresponding sample collection time points and MLSTs. Download Table S1, DOCX file, 0.1 MB. Copyright © 2018 Bevan et al. 2018 Bevan et al. This content is distributed under the terms of the Creative Commons Attribution 4.0 International license.

The only data on the distribution of the E. coli STs in South Asia come from three centers: Pune (19, 20), Kolkata (21), and Islamabad (22). Roy et al. found that 6/67 isolates causing bacteremia on a neonatal intensive care unit were ST131 using a PCR-based method: no MLST was carried out (21). Hussain et al. also used a PCR-based approach only, finding that 16/23 ESBL-producing E. coli isolates identified in Pune were ST131 (19). Ranjan et al. determined the MLST of only 8 clinical isolates from Pune, India, finding that 2/8 were E. coli ST131, with no single ST predominating (20). Zahra et al. surveyed the MLSTs of 110 E. coli isolates sampled from human sewage across multiple locations in Islamabad, Pakistan (22). Zahra and colleagues found 24 STs, with ST394, ST10, and ST648 predominating, with only 1/110 being ST131 (22). Apart from ST10, the other dominant STs seen by Zahra et al. were not seen in our study. It is notable that ST131 was a rare occurrence in the Islamabad sewage network, contrasting with the prevalence of ST131 CTX-M-EC in our study. This may be due to ST131 E. coli being an exceptional colonizer of the human fecal microbiome, rather than of the sewage environment in Islamabad. Due to the paucity of data in South Asia, our study provides a much-needed “snapshot” of E. coli strains originating from the region.

Dureja et al. obtained fecal samples from 102 healthy adults in Chandigarh, India, during 2012 to 2013 and used PCR to determine the phylogenetic groups (23). Dureja and colleagues found that the most common E. coli phylogenetic groups were A (54%, 55/102) and B1 (24%, 24/102), with 9/15 CTX-M-15-producing E. coli strains belonging to PG-A (23). This dominance of PG-A is in keeping with previous work which showed that PG-A tends to predominate in the human fecal microbiome in tropical regions compared to temperate regions (24).

Relatively few studies have assessed the E. coli MLSTs from the human fecal microbiome identified after travel, but all found there to be a wide range of ESBL-producing E. coli MLSTs acquired through travel (12, 13, 15). Pires and colleagues are the only group to have assessed the colonization with particular E. coli STs before and after travel, showing that travel from Switzerland to India results in polyclonal colonization with CTX-M-producing E. coli with a mean of two STs and a range of 1 to 5 (13). Kuenzli et al. found that in a representative population of 34 CTX-M-EC isolates from an undescribed number of volunteers acquired after travel to India, there were 24 different STs (12). Paltansing et al. found 86 different STs from 146 ESBL-producing E. coli isolates from 133 travelers but did not describe the specific STs acquired from South Asia or India (15). In our study, we identified 26 different STs from 198 post-travel CTX-M-EC isolates, collected from 15 volunteers (Table S1).

### CTX-M-EC clones are maintained after travel.

The mean duration of carriage of CTX-M-EC was 113 days, with a median of 60 days. This carriage duration must be considered an underestimate, because 8/16 volunteers remained CTX-M-EC carriers at the last fecal sampling point (Table 1). Thirty-one percent (5/16) of volunteers were known to be CTX-M-EC positive at 6 months after travel. The volunteers with the longest duration of carriage of CTX-M-EC were colonized with ST131 (PG-B2) (11 months, volunteer 8), ST405 (PG-D) (9 months, volunteer 3), and ST648 (PG-B1) (8 months, volunteer 8). Considering all strains which colonized the fecal microbiome for the longest duration, there was no predominance of any particular ST or PG.

Arcilla et al. found 11.3% (68/601) were persistently colonized with the same ESBL-producing organism at 6 months post-travel, with a median post-travel duration of colonization of 30 days, but did not report a subgroup analysis on the duration of carriage for 79 travelers who went to India (6). Another study supporting this finding was that of Pires et al., who found a 40% colonization rate (6/15 travelers), 6 months after return from India (13). Ruppé et al. also found that 7.2% (8/111) of ESBL-carriage-positive travelers were still carriers at 6 months after travel to Asia, with no specific country data (11). Moreover, Ruppé et al. reported that travel to Asia is significantly associated with a longer duration of carriage than travel to Africa or Latin America (11). A more recent Canadian study found that 15/70 (21%) travelers to South Asia who acquired ESBL-producing E. coli, were still colonized 6 months after return to Canada (10).

Eight volunteers in our study provided stool samples at two or more time points after travel. All these volunteers carried CTX-M-EC of the same ST in stool samples at different time points. By SNP typing, in 8/8 volunteers the isolates found in separate fecal samples are <10 SNPs apart, suggesting stable colonization with the same strain over several months (Table 3). Four volunteers had post-travel CTX-M-EC isolates which were indistinguishable (0 SNPs) between separate post-travel fecal samples collected at different time points (Table 3).

**TABLE 3:** SNP typing of post-travel CTX-M-EC

Strain^*a*^	Sample collection point (days post-travel)	MLST	Phylogenetic group
3b3	7	1193^*b*^	B2
3b4	7	1193^0^	B2
3c3	28	1193^0^	B2
3d1	56	1193^63^	B2
3d2	56	1193^52^	B2
3d3	56	1193^0^	B2
3d4	56	1193^63^	B2
4a6	7	69^*b*^	D
4b1	30	69^5^	D
4b2	30	69^4^	D
4b3	30	69^4^	D
4b4	30	69^4^	D
4c1	49	69^4^	D
4c4	49	69^4^	D
4e1	152	69^4^	D
4e2	152	69^5^	D
4e4	152	69^4^	D
5.2a	4 post-Uzbekistan	38^*b*^	D
5.2b	4 post-Uzbekistan	38^2^	D
5.2c	4 post-Uzbekistan	38^2^	D
5.2e	4 post-Uzbekistan	38^2^	D
5.2f	4 post-Uzbekistan	38^2^	D
5a1	3 post-India, 35 post-Uzbekistan	48^*d*^	A
5a2	3 post-India, 35 post-Uzbekistan	^46^48	A
5a3	3 post-India, 35 post-Uzbekistan	^42^48	A
5a4	3 post-India, 35 post-Uzbekistan	^39^48	A
5b1	8 post-India	^48^48	A
5b3	8 post-India	^33^48	A
5b4	8 post-India	^26^48	A
5c4	28 post-India	^46^48	A
5e3	91 post-India	38^6^	D
5e4	91 post-India	38^6^	D
5f3	183 post-India	^12765^48	A
8b2	16	10^*b*^	A
8b3	16	10^1^	A
8b4	16	10^1^	A
8c1	40	10^2^	A
8c2	40	10^43^	A
8c3	40	10^2^	A
8c4	40	10^2^	A
8d1	58	10^1^	A
8d2	58	10^13331^	A
8d4	58	10^13323^	A
9b1	14	2617^19^	F
9b2	14	2617^*b*^	F
9c1	35	2617^9^	F
9c2	35	2617^5^	F
9c3	35	2617^7^	F
9c4	35	2617^1^	F
9d1	91	2617^2^	F
9d3	91	2617^2^	F
9d4	91	2617^7^	F
9e1	152	2617^4^	F
9e2	152	2617^0^	F
9e3	152	2617^6^	F
9e4	152	2617^1^	F
9f1	210	2617^3^	F
9f2	210	2617^7^	F
9f3	210	2617^5^	F
9f4	210	2617^4^	F
12a1	3	43^5^	A
12a2	3	43^*b*^	A
12a3	3	43^0^	A
12a4	3	43^0^	A
12b1	21	43^0^	A
12b2	21	43^0^	A
12b3	21	43^0^	A
12b4	21	43^1^	A
12c1	29	43^0^	A
12c2	29	43^0^	A
12c3	29	43^0^	A
12c4	29	43^0^	A
12d1	56	43^1^	A
12d2	56	43^3^	A
12d3	56	43^2^	A
12d4	56	43^2^	A
12e1^*b*^	115	43^0^	A
12e2	115	43^0^	A
12e3	115	43^1^	A
12e4	115	43^1^	A
16b2	40	131^*b*^	B2
16c1	91	131^4^	B2
16c2	91	131^3^	B2
16c4	91	131^2^	B2
16e1	179	131^3^	B2
16e2	179	131^4^	B2
16e3	179	131^3^	B2
16e4	179	131^6^	B2
20a3	7	162^5^	NG^*c*^
20a4	7	162^*b*^	NG
20b1	42	162^0^	NG
20b2	42	162^0^	NG
20b3	42	162^0^	NG
20b4	42	162^0^	NG

a Isolate naming system: e.g., 3b3, where “3” stands for volunteer 3, “b” stands for the second post-travel fecal sample, and the following digit “3” stands for the third colony pick from the sample.

b Reference strain for SNP comparisons for any given volunteer. SNPs are displayed as superscripts after the ST.

c NG, no phylogenetic group assigned to the Warwick MLST.

d Second reference strain where 2 STs are present (volunteer 5). SNPs are displayed as superscripts before the ST.

Given the diversity within the larger STs of E. coli, it is not surprising that we also found colonization with completely different strains defined by WGS of the same MLST group. Volunteer 8 carried two ST10 populations after travel which were 13,323 SNPs apart (Table 3).

Volunteer 5 is particularly notable as being the only volunteer to make a trip outside South Asia before the trip to India. She first traveled to Uzbekistan for 10 days and then returned to the United Kingdom for 30 days. This was followed by a trip to India for 14 days and then return to the United Kingdom. Volunteer 5 submitted one sample pre-Uzbekistan (5.1), and no strains found were subsequently isolated (Table S1). Sample 5.2 was collected 4 days after return from Uzbekistan (CTX-M-EC, ST38), and sample 5a1 was collected 3 days after the volunteer returned from India (ST48 CTX-M-EC only). Sample 5e4 was collected 12 weeks after return from India (ST38 CTX-M-EC only) (Table 3). The ST38 isolates in fecal samples 5.2 and 5e4 are only 6 SNPs different, suggesting that the same CTX-M-EC strain remained as a colonizer within the fecal microbiome post-Uzbekistan, during the trip to India, and was again maintained on return to the United Kingdom (Table 3). An additional ST48 strain (5f3) was identified from sample 5f (6 months after returning from India): this strain, 5f3, was 12,765 SNPs different from the ST48 strains from earlier post-India samples (Table 3). Strain 5f3 is most likely to be a travel-acquired strain which was not detected in the earlier post-travel samples.

Pires and colleagues used MLST and repetitive palindromic PCR (RepPCR) to demonstrate that CTX-M-EC strains detected immediately post-travel are detected again at 3 and 6 months post-travel, with the same RepPCR fingerprint (13). However, Pires did not carry out SNP typing to prove that the same strains are carried over time, nor did they present the MLST or RepPCR profiles of pre-travel non-CTX-M-EC.

The polyclonal nature of CTX-M-producing E. coli acquisition in our study suggests multiple acquisition events during travel, which is in keeping with exposure to food and water supplies contaminated with fecal bacteria. Carriage of the same CTX-M-EC clones—often for several months after return to the United Kingdom—shows that the acquired strains are not easily displaced and suggests stable colonization in the fecal microbiome in the absence of antibiotic selective pressure.

### Non-CTX-M-producing E. coli strains acquired during travel co-colonize the GI tract alongside CTX-M-EC strains after acquisition during travel.

To determine whether fecal samples containing CTX-M-EC co-colonize the fecal microbiome with CTX-M non-producers, a toothpicking experiment was undertaken with fecal samples from a representative selection of volunteers (eight) who were CTX-M-EC positive. One hundred individual colonies from stool culture plates were individually spotted onto antibiotic-free agar and then onto cefotaxime-containing agar. Picking 100 colonies per fecal sample allows the isolation of the major and minority E. coli strains in the sample (25). The cefotaxime-sensitive strains were then tested for the absence of CTX-M-EC using CTX-M grouping PCR.

**FIG 3:**
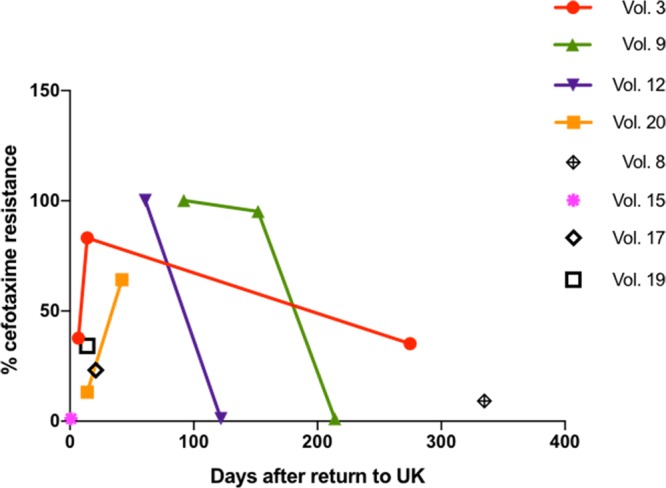
Proportion of cefotaxime-resistant strains in post-travel fecal samples containing CTX-M-EC. Vol., volunteer.

The results showed that the proportion of cefotaxime-resistant E. coli in fecal samples known to contain CTX-M-EC varied considerably between volunteers and at different time points for the same volunteer (Fig. 3). In fecal samples collected from the same volunteer that were analyzed using toothpicking over time, the proportion of cefotaxime-resistant strains did not remain stable (Fig. 3). In 3/4 volunteers for whom we undertook toothpicking on multiple fecal samples, the proportion of cefotaxime-resistant strains diminished over time (Fig. 3), probably reflecting competition from non-CTX-M-EC and lack of reexposure to CTX-M-EC through ingestion in the United Kingdom. In 7/7 cases where non-CTX-M-EC isolates were detected by toothpicking in stool samples that contained CTX-M-EC, WGS revealed that these strains were not related to either the endogenous non-CTX-M-EC present before travel or the CTX-M-EC acquired during travel (Table 2).

The present study is thus the first to demonstrate co-colonization of third-generation-cephalosporin-sensitive E. coli in the fecal microbiome containing CTX-M-producing strains. There are two possibilities underlying this phenomenon: (i) gut colonization during travel includes CTX-M producers and CTX-M non-producers and (ii) the CTX-M non-producers were acquired after return to the United Kingdom and were detected in stool samples co-colonizing with travel-acquired CTX-M producers. It is also possible that both scenarios take place. However, in 7/7 toothpicking experiments, the third-generation-cephalosporin-sensitive E. coli STs detected were different from both the pre-travel CTX-M-free strains and the post-travel CTX-M producers. Thus, our data suggest that travel results in acquisition of both CTX-M producers and non-producers (Table 2), providing further evidence of probable multiple episodes of E. coli ingestion and colonization via food and drink during travel. Sampling volunteers while overseas, which was beyond the scope of our study, would be required to confirm this hypothesis. This would allow determination of how long it took for each volunteer to acquire CTX-M-EC and non-CTX-M E. coli during their trip.

The toothpicking approach was the most effective way in which to (i) determine the diversity of E. coli strains, including determining the minority strains present in a sample of 100 colony picks, and (ii) allow linkage of CTX-M carriage or noncarriage with specific E. coli STs. Alternative methods, such as the use of shotgun metagenomic sequencing of fecal samples, would not allow the same resolution for the analysis of E. coli clonal dynamics as our toothpicking approach, in particular, assigning *bla*_CTX-M_ genes to specific strains. In addition, shotgun metagenomic analysis has relatively low sensitivity in detecting minority bacterial populations, compared to culture (26).

### Pre-travel resident E. coli strains persist as a minority population in the fecal microbiome.

For seventeen volunteers, non-CTX-M-EC populations were sequenced before and after travel. In the case of seven volunteers who had acquired and then lost CTX-M-EC, we detected the same non-CTX-M-EC MLSTs before and after travel. We used SNP typing to define whether these strains were the same clonal lineage of non-CTX-M-EC before versus after travel. In 5/7 volunteers we found near-indistinguishable strains (<20 SNPs) before and after travel (Table 4). All five volunteers also acquired and lost co-colonizing CTX-M-EC. This is a new finding: pre-travel E. coli strains are not completely displaced from the fecal microbiome but are maintained as a minority population throughout travel and become detectable again as a majority population after travel (Table 4).

**TABLE 4:** SNP comparisons of pre- and post-travel non-CTX-M-producing *E. coli* for volunteers with the same MLSTs before and after travel

Volunteer	Isolate	Sample collection point^*a*^ (days pre- or post-travel)	MLST	Phylogenetic group
1	1_1a	**10 pre**	131^*b*^	B2
	1_1b	**10 pre**	131^5^	B2
	1_1c	**10 pre**	131^1^	B2
	1_1d	**10 pre**	131^19^	B2
	1_2a	**5 pre**	131^17^	B2
	1_2b	**5 pre**	131^18^	B2
	1_2c	**5 pre**	131^6^	B2
	1_e_1	*122 post*	131^3^	B2
	1_e_2	*122 post*	131^6^	B2
	1_e_3	*122 post*	131^2^	B2
6	6_1c	**42 pre**	73^17^	B2
	6_1e	**42 pre**	73^14^	B2
	6_2a	**28 pre**	73^77^	B2
	6_2b	**28 pre**	73^*b*^	B2
	6_2c	**28 pre**	73^14^	B2
	6_2d	**28 pre**	73^22^	B2
	6_3a	**1 pre**	73^23^	B2
	6_3b	**1 pre**	73^17^	B2
	6_3c	**1 pre**	73^27^	B2
	6_3d	**1 pre**	73^17^	B2
	6_3e	**1 pre**	73^20^	B2
	6_3f	**1 pre**	73^88^	B2
	6c2	*42 post*	73^1^	B2
	6c4	*42 post*	73^30^	B2
	6d1	*180 post*	73^24^	B2
	6d2	*180 post*	73^24^	B2
	6d3	*180 post*	73^5^	B2
	6d4	*180 post*	73^20^	B2
7	7_1a	**60 pre**	10^*b*^	A
	7_1b	**60 pre**	10^2^	A
	7_1c	**60 pre**	10^2^	A
	7_1d	**60 pre**	10^1^	A
	7_1e	**60 pre**	10^0^	A
	7_1f	**60 pre**	10^5^	A
	7_3b	**7 pre**	10^2^	A
	7_3c	**7 pre**	10^3^	A
	7_3d	**7 pre**	10^4^	A
	7_3e	**7 pre**	10^2^	A
	7_3f	**7 pre**	10^2^	A
	7a1	*5 post*	10^18143^	A
	7b1	*10 post*	10^18141^	A
	7b2	*10 post*	10^18124^	A
8	8.2a	**1 pre**	10^*b*^	A
	8.2b	**1 pre**	10^5^	A
	8.2d	**1 pre**	10^2^	A
	8.2e	**1 pre**	10^17^	A
	8.2f	**1 pre**	10^1^	A
	8b2	*16 post*	10^17093^	A
	8b3	*16 post*	10^17084^	A
	8b4	*16 post*	10^17085^	A
	8c1	*40 post*	10^17085^	A
	8c2	*40 post*	10^17087^	A
	8c3	*40 post*	10^17084^	A
	8c4	*40 post*	10^17088^	A
	8d1	*58 post*	10^17084^	A
	8d2	*58 post*	10^13306^	A
	8d4	*58 post*	10^13307^	A
	8d5	*58 post*	10^17084^	A
15	15_1c	**28 pre**	10^*b*^	A
	15_1d	**28 pre**	10^6^	A
	15_2c	**14 pre**	10^10^	A
	15_2d	**14 pre**	10^54^	A
	15c1	*150 post*	10^9^	A
	15c2	*150 post*	10^7^	A
	15c3	*150 post*	10^23^	A
	15c4	*150 post*	10^14^	A
17	17_2a	**3 pre**	131^*b*^	B2
	17_2b	**3 pre**	131^18^	B2
	17_2c	**3 pre**	131^12^	B2
	17_2d	**3 pre**	131^11^	B2
	17_2e	**3 pre**	131^27^	B2
	17_2f	**3 pre**	131^8^	B2
	17c1	*84 post*	131^16^	B2
	17c2	*84 post*	131^29^	B2
	17c3	*84 post*	131^17^	B2
	17c4	*84 post*	131^45^	B2
20	20_1b	**10 pre**	10^13568^	A
	20_1c	**10 pre**	10^13553^	A
	20_2a	**4 pre**	10^14956^	A
	20bpp	*42 post*	10^*b*^	A
	20f1	*120 post*	10^10^	A
	20g1	*183 post*	10^24^	A
	20g2	*183 post*	10^9^	A
	20g3	*183 post*	10^9^	A
	20g4	*183 post*	10^31^	A

a Sample collection point includes only fecal samples where the *E. coli* sequence type displayed for that volunteer was detected. For the full list of isolates, see Table S1. Boldface indicates pre-travel fecal samples, and italic indicates post-travel.

b Reference strain for SNP comparisons for any given volunteer. SNPs are displayed as superscripts after the ST.

Previous research into the effect of travel on the composition of the fecal microbiome using metagenomic sequencing showed that in Swedish travelers going to India or Central Africa, there is a spike in abundance of the *Proteobacteria* phylum after travel (27). Increasing abundance of *Proteobacteria* may result from low-level inflammation as a result of change in diet, low-level gut inflammation due to travel, and gastroenteritis due to infecting bacteria and parasites (27), therefore allowing colonization with CTX-M-EC.

A metagenomic study undertaken by David and colleagues showed that the human fecal microbiome returns to its pre-travel state after perturbation resulting from travel (28). David et al. sequenced the fecal microbiome over 1 year, in an individual who traveled from the United States to Southeast Asia for a period of 51 days, and showed that travel results in a swing to *Bacteroidetes* and *Proteobacteria*, which is reversed on returning to the home environment (28).

A small number of studies have considered the E. coli clonal dynamics of travel-acquired strains over time (13–15). Pires et al. (13) found 3/15 volunteers who were CTX-M positive before travel, and in 1/3 cases, the ST648 CTX-M-14-producing strain found before travel was also detected at 3 months after travel and was presumably the same pre-travel strain. Similarly, Vading et al. used Rep-PCR to show that four volunteers carried the same PG-B2 ESBL-producing *Enterobacteriaceae* before and after travel (14). Moreover, Paltansing et al. showed that 4/7 volunteers who were CTX-M positive pre-travel had the same CTX-M-strain genotype and ST combination detected in fecal samples after travel (15). Based on the findings from Pires et al., Vading et al., and Paltansing et al., it is reasonable to suggest that pre-travel CTX-M-EC strains often persist throughout the period of travel and are detectable in post-travel fecal samples. However, unlike the present study, none of these studies did SNP typing of strains nor did they determine diversity of pre-travel non-ESBL E. coli, so definitive evidence of pre-travel strain persistence after travel was not provided. Our study is the first to provide evidence from MLST and SNP typing that indistinguishable cefotaxime-sensitive strains (non-CTX-M-EC) are present before and after travel to South Asia.

### Conclusions.

This study has confirmed the high rates of acquisition of CTX-M-EC by travelers from countries of low CTX-M-EC endemicity to areas with high community prevalence of CTX-M-EC and is the first such study to recruit healthy travelers prospectively from the United Kingdom. We show that travelers usually acquire multiple E. coli sequence types carrying *bla*_CTX-M_, the same CTX-M-EC clonal lineages are carried in the gut after return to the United Kingdom, and the post-travel fecal microbiome often contains both CTX-M-EC and non-CTX-M-EC.

There are some limitations around the population recruited in this study, leading to possible selection bias. The volunteers were all university or hospital employees or students and are not representative of the United Kingdom population with respect to age or socioeconomic level. In addition, 5/16 travelers who acquired CTX-M-EC did medical elective placements in hospitals in India or Sri Lanka. Therefore, they may have acquired strains from these institutions, rather than from community exposure.

A further limitation is that we did not include non-E. coli *Enterobacteriaceae* in this study. We found that *Klebsiella* and *Enterobacter* species actually made up a very small number of isolates cultured on the chromogenic agar; therefore, we chose to focus on the predominant E. coli. It must also be noted that in previous studies, E. coli was the main carrier of *bla*_CTX-M_ among the *Enterobacteriaceae* (3, 6).

It must also be noted that we did not investigate the specific CTX-M-EC acquisition events which occurred during travel. This would involve sampling while outside the United Kingdom and was beyond the scope of the present study.

We have provided novel data on the dynamics of colonization with CTX-M-EC and non-CTX-M-EC after travel. Our data provides evidence that humans acquire both CTX-M-EC and non-CTX-M-EC after travel to South Asia, while a minority population of pre-travel E. coli strains re-emerge as the dominant E. coli population after CTX-M-EC is lost. The loss of CTX-M-EC after travel, albeit sometimes up to a year post-travel, may be due to the competition from preexisting non-CTX-M E. coli. The manipulation of non-pathogenic, non-ESBL-EC in order to displace virulent antibiotic-resistant strains in community and health care settings (including carbapenemase-producing *Enterobacteriaceae* and *mcr-1*-producing strains) is an avenue worth exploring in the battle against the spread of antimicrobial resistance in *Enterobacteriaceae*.

## MATERIALS AND METHODS

### Volunteer recruitment and inclusion/exclusion criteria.

Volunteers were recruited from the student and staff members of the University of Birmingham and University Hospitals Birmingham Heartlands Hospital, using posters and targeted emails and via Twitter in the period March 2015 to June 2016. Individuals planning travel to South Asia contacted E.R.B. by email or telephone, at which point they were excluded from taking part if they reported previous travel to South Asia in the previous three months or if they had a significant long-term chronic disease. South Asia includes Afghanistan, Bangladesh, Bhutan, India, Maldives, Nepal, Pakistan, and Sri Lanka (29), but only volunteers planning travel to Bangladesh, India, Pakistan, and Sri Lanka were encountered. Written informed consent was obtained from all volunteers who took part.

### Laboratory methodology: sample collection, culture, and identification.

Volunteers were asked to provide at least 5 g of feces and were asked to provide a specimen as close to the time of sample submission as possible. Volunteers were then met in person by E.R.B., and samples were stored for no more than 24 h at 4°C before culture.

The following culture-based ESBL-EC isolation strategies were then undertaken. (i) A sterile swab was used to inoculate samples onto Oxoid ESBL brilliance agar, which was incubated at 37°C for 24 to 48 h. (ii) Additional chromogenic agar (Oxoid UTI brilliance agar) supplemented with a cefpodoxime disc (10 µg/ml) was inoculated with serial dilutions of stool culture, to allow isolation of single colonies after 24-h incubation. (iii) Brain heart infusion (BHI) broth supplemented with a cefpodoxime disc (10 µg/ml) was also inoculated with a sterile swab loaded with feces, incubated at 37°C for 24 h. A 10-µl loop of the overnight BHI was then subcultured onto a fresh UTI chromogenic agar plate supplemented with a cefpodoxime disc and incubated for a further 24 h.

Putative ESBL-EC strains were identified as blue or pink colonies on ESBL agar, and pink or clear colonies within the cefpodoxime disc zone (or outside the zone in pre-travel samples) on UTI medium before and after broth enrichment. Each colony pick was confirmed to be E. coli using matrix-assisted laser desorption–ionization time of flight mass spectroscopy (MALDI-TOF MS). Colonies with differing morphology were always picked and identified in this way.

PCR was used as a screening method before putative ESBL producers were subjected to WGS. Primers for multiplex PCR as performed previously were used to identify the three most common CTX-M groups: group 1, group 2, and group 9 (30). PCR was carried out only on isolates which had grown either on ESBL agar or from within the zones of cefpodoxime on nonselective UTI agar. PCR amplimers were visualized using 1% agarose gel electrophoresis.

### Toothpicking experiments.

In order to characterize the cefotaxime-sensitive population of E. coli within stool samples containing CTX-M-positive post-travel E. coli, a representative collection of post-travel stool samples were subjected to toothpicking experiments. Oxoid UTI brilliance agar was used to culture E. coli (pink colonies) directly from stool samples. A sterile wire was used to pick individual E. coli colonies from the chromogenic plate. Each colony was then lightly “scored” (2 to 3 mm) onto antibiotic-free LB agar and onto LB agar containing cefotaxime (4 µg/ml). One hundred colonies were picked from each stool sample tested.

Isolates which grew on the antibiotic-free plate but failed to grow on the cefotaxime plate were enumerated.

### Whole-genome sequencing of isolates.

Automated DNA extraction and whole-genome sequencing were undertaken by MicrobesNG at the University of Birmingham. Sequencing was performed using HiSeq 2500 with 250-bp paired-end reads. QC was performed using QUAST (31). All isolates were assembled using SPAdes (32) and annotated using Prokka (33).

### Bioinformatic analyses.

All sequenced E. coli isolates were subjected to analysis, by determining the multilocus sequence type (MLST) (34). The E. coli Achtman MLST scheme (https://enterobase.warwick.ac.uk/species/index/ecoli) of each strain was determined by inputting contig files and running the mlst program () (35). The E. coli phylogenetic group was derived MLST data, as previously described (36).

### Identifying E. coli clones from WGS data.

Illumina sequence reads and assembled draft genomes were used to determine the phylogenetic relationships of selected strains. In addition to obtaining the MLST of each E. coli strain in the study, a core genome alignment of all strains was also undertaken, using Parsnp (37).

Further SNP typing was undertaken to ascertain the relationship between identical STs (i.e., the number of SNPs between E. coli strains). Snippy was used to find SNPs between a chosen reference genome and a set of sequence reads, thus creating a core genome SNP alignment, as described at https://github.com/tseemann/snippy. SNP distances between each isolate in the phylogeny were determined using snp-dists, which produces an output in the form of a table matrix (https://github.com/tseemann/snp-dists).

### Ethical approval.

This study was approved by the South Birmingham Research Ethics Committee (reference 15/WM/0037).
