# Supplementary material for: Acquisition and Loss of CTX-M-Producing and Non-Producing Escherichia coli in the Fecal Microbiome of Travelers to South Asia
Source: mBio. 2018 Dec 11;9(6):e02408-18. doi: 10.1128/mBio.02408-18 (PMC6299485; doi:10.1128/mBio.02408-18)
Supplement: TABLE S1 [file mbo006184217st1.docx]

| **Table S1**. Isolates sequenced pre and post travel with corresponding sample collection time points and MLSTs | | | |
| --- | --- | --- | --- |
|  |  |  |  |
| **Isolate ID** | **Sample time-point** | **MLST** | **Phylogenetic group** |
| 1.1a | 10 days pre | 131 | B2 |
| 1.1b |  | 131 | B2 |
| 1.1c |  | 131 | B2 |
| 1.1d |  | 131 | B2 |
| 1.1e |  | NS |  |
| 1.1f |  | NS |  |
| 1.2a | 5 days pre | 131 | B2 |
| 1.2b |  | 131 | B2 |
| 1.2c |  | 131 | B2 |
| 1.2d |  | NS |  |
| 1.2e |  | NS |  |
| 1.2f |  | NS |  |
| 1a1 | 4 days post | NT |  |
| 1a2 |  | 349 | D |
| 1a3 |  | 349 | D |
| 1a4 |  | 349 | D |
| 1bs | 19 days post | 394 | D |
| 1cs | 31 days post | 69 | D |
| 1.e.1 | 122 days post | 131 | B2 |
| 1.e.2 |  | 131 | B2 |
| 1.e.3 |  | 131 | B2 |
| 1.e.4 |  | NT |  |
| 3.1a | 28 days pre | 69 | D |
| 3.1b |  | 941 | B2 |
| 3.1c |  | 941 | B2 |
| 3.1d |  | 69 | D |
| 3.1e |  | 69 | D |
| 3.1f |  | 69 | D |
| 3.2a | 14 days pre | 69 | D |
| 3.2b |  | 69 | D |
| 3.2c |  | 69 | D |
| 3.2d |  | 69 | D |
| 3.2e |  | 69 | D |
| 3.2f |  | 2008 | NG |
| 3.3a | 3 days pre | 69 | D |
| 3.3b |  | 69 | D |
| 3.3c |  | 69 | D |
| 3.3d |  | 69 | D |
| 3.3e |  | 69 | D |
| 3.3f |  | 69 | D |
| 3a1 | 7 days post | 43 | A |
| 3a2 |  | 43 | A |
| 3b2 | 7 days post | 38 | D |
| 3b3 |  | 1193 | B2 |
| 3b4 |  | 1193 | B2 |
| 3c1 | 28 days post | 38 | D |
| 3c3 |  | 1193 | B2 |
| 3d1 | 56 days post | 1193 | B2 |
| 3d2 |  | 1193 | B2 |
| 3d3 |  | 1193 | B2 |
| 3d4 |  | 1193 | B2 |
| 3.e.1 | 84 days post | 405 | D |
| 3.e.2 |  | 405 | D |
| 3.e.3 |  | 38 | D |
| 3.e.4 |  | 405 | D |
| 3f1 | 274 days post | 405 | D |
| 3f2 |  | 405 | D |
| 3f3 |  | 405 | D |
| 3f4 |  | 405 | D |
| 4.1a | 14 days pre | 10 | A |
| 4.1b |  | 10 | A |
| 4.1c |  | 10 | A |
| 4.1d |  | 10 | A |
| 4.1e |  | 10 | A |
| 4.1f |  | 10 | A |
| 4.2a | 7 days pre | 10 | A |
| 4.2b |  | 10 | A |
| 4.2c |  | 10 | A |
| 4.2d |  | 10 | A |
| 4.2e |  | 10 | A |
| 4.2f |  | 10 | A |
| 4a1 | 7 days post | 2732 | NG |
| 4a2 |  | 2732 | NG |
| 4a3 |  | 2732 | NG |
| 4a4 |  | 2732 | NG |
| 4a5 |  | 2732 | NG |
| 4a6 |  | 69 | D |
| 4b1 | 30 days post | 69 | D |
| 4b2 |  | 69 | D |
| 4b3 |  | 69 | D |
| 4b4 |  | 69 | D |
| 4c1 | 49 days post | 69 | D |
| 4c2 |  | NT |  |
| 4c3 |  | NT |  |
| 4c4 |  | 69 | D |
| 4d1 | 91 days post | 648 | B1 |
| 4d2 |  | 648 | B1 |
| 4d3 |  | 648 | B1 |
| 4d4 |  | 648 | B1 |
| 4.e.1 | 152 days post | 69 | D |
| 4.e.2 |  | 69 | D |
| 4.e.3 |  | NT |  |
| 4.e.4 |  | 69 | D |
| 5.1a | 7 days Pre-Uzbekistan | 452 | B2 |
| 5.1b |  | 452 | B2 |
| 5.1c |  | 452 | B2 |
| 5.1d |  | 452 | B2 |
| 5.1e |  | 452 | B2 |
| 5.1f |  | 452 | B2 |
| 5.2a | 4 days post Uzbek. | 38 | D |
| 5.2b |  | 38 | D |
| 5.2c |  | 38 | D |
| 5.2d |  | 405 | D |
| 5.2e |  | 38 | D |
| 5.2f |  | 38 | D |
| 5.3a | 29 days post Uzbek. | 405 | D |
| 5.3b |  | 405 | D |
| 5.3c |  | 405 | D |
| 5.3d |  | 131 | B2 |
| 5.3e |  | 131 | B2 |
| 5.3f |  | 131 | B2 |
| 5a1 | 3 days post India | 48 | A |
| 5a2 |  | 48 | A |
| 5a3 |  | 48 | A |
| 5a4 |  | 48 | A |
| 5b1 | 8 days post India | 48 | A |
| 5b2 |  | NT |  |
| 5b3 |  | 48 | A |
| 5b4 |  | 48 | A |
| 5c1 | 28 days post India | 10 | A |
| 5c2 |  | 10 | A |
| 5c3 |  | 10 | A |
| 5c4 |  | 48 | A |
| 5d1 | 60 days post India | 10 | A |
| 5d2 |  | 10 | A |
| 5d3 |  | 10 | A |
| 5d4 |  | 10 | A |
| 5.e.1 | 91 days post India | 6338 | NG |
| 5.e.2 |  | 6338 | NG |
| 5.e.3 |  | 38 | D |
| 5.e.4 |  | 38 | D |
| 5f1 | 183 days post India | 88 | C |
| 5f2 |  | NT |  |
| 5f3 |  | 48 | A |
| 5f4 |  | 88 | C |
| 5g1 | 244 days post India | 3478 | A |
| 5g2 |  | 3478 | A |
| 5g3 |  | 3478 | A |
| 5g4 |  | 3478 | A |
| 5H1 | 364 days post India | 10 | A |
| 5H2 |  | NT |  |
| 5H3 |  | 10 | A |
| 5H4 |  |  |  |
| 6.1a | 42 days pre | 69 | D |
| 6.1b |  | 69 | D |
| 6.1c |  | 73 | B2 |
| 6.1d |  | 69 | D |
| 6.1e |  | 73 | B2 |
| 6.1f |  | 69 | D |
| 6.2a | 28 days pre | 73 | B2 |
| 6.2b |  | 73 | B2 |
| 6.2c |  | 73 | B2 |
| 6.2d |  | 73 | B2 |
| 6.3a | 1 day pre | 73 | B2 |
| 6.3b |  | 73 | B2 |
| 6.3c |  | 73 | B2 |
| 6.3d |  | 73 | B2 |
| 6.3e |  | 73 | B2 |
| 6.3f |  | 73 | B2 |
| 6a1 | 4 days post | 450 | NG |
| 6a2 |  | 450 | NG |
| 6a3 |  | 450 | NG |
| 6a4 |  | 450 | NG |
| 6b1 | 21 days post | NT |  |
| 6b2 |  | NT |  |
| 6b3 |  | NT |  |
| 6b4 |  | NT |  |
| 6c1 | 42 days post | 2221 | B1 |
| 6c2 |  | 73 | B2 |
| 6c3 |  | 450 | NG |
| 6c4 |  | 73 | B2 |
| 6d1 | 180 days post | 73 | B2 |
| 6d2 |  | 73 | B2 |
| 6d3 |  | 73 | B2 |
| 6d4 |  | 73 | B2 |
| 7.1a | 60 days pre | 10 | A |
| 7.1b |  | 10 | A |
| 7.1c |  | 10 | A |
| 7.1d |  | 10 | A |
| 7.1e |  | 10 | A |
| 7.1f |  | 10 | A |
| 7.2a | 29 days pre | NT |  |
| 7.2b |  | 69 | D |
| 7.2c |  | NT |  |
| 7.2d |  | NT |  |
| 7.2e |  | NT |  |
| 7.2f |  | NT |  |
| 7.3a | 7 days pre | 69 | D |
| 7.3b |  | 10 | A |
| 7.3c |  | 10 | A |
| 7.3d |  | 10 | A |
| 7.3e |  | 10 | A |
| 7.3f |  | 10 | A |
| 7a1 | 5 days post | 10 | A |
| 7a2 |  | 6438 | NG |
| 7a3 |  | 6438 | NG |
| 7a4 |  | 6438 | NG |
| 7b1 | 10 days post | 10 | A |
| 7b2 |  | 10 | A |
| 7b3 |  | NT |  |
| 7b4 |  | 43 | A |
| 7c2 | 28 days post | 200 | B1 |
| 7c3 |  | 200 | B1 |
| 7c4 |  | 200 | B1 |
| 7d1 | 60 days post | 200 | B1 |
| 7d2 |  | 200 | B1 |
| 7d3 |  | 200 | B1 |
| 7d4 |  | 200 | B1 |
| 7.e.1 | 122 days post | 450 | NG |
| 7f2 | 245 days post | 3036 | NG |
| 7f4 |  | 69 | D |
| 8.1a | 7 days pre | 69 | D |
| 8.1b |  | 1163 | NG |
| 8.1c |  | 69 | D |
| 8.1f |  | 2712 | D |
| 8.2a | 1 day pre | 10 | A |
| 8.2b |  | 10 | A |
| 8.2c |  | NT |  |
| 8.2d |  | 10 | A |
| 8.2e |  | 10 | A |
| 8.2f |  | 10 | A |
| 8a1 | 10 days post | 448 | B1 |
| 8a2 |  | 448 | B1 |
| 8a3 |  | 448 | B1 |
| 8a4 |  | 448 | B1 |
| 8b1 | 16 days post | 648 | B1 |
| 8b2 |  | 10 | A |
| 8b3 |  | 10 | A |
| 8b4 |  | 10 | A |
| 8c1 | 40 days post | 10 | A |
| 8c2 |  | 10 | A |
| 8c3 |  | 10 | A |
| 8c4 |  | 10 | A |
| 8d1 | 58 days post | 10 | A |
| 8d2 |  | 10 | A |
| 8d3 |  | 648 | B1 |
| 8d4 |  | 10 | A |
| 8.e.1 | 120 days post | 648 | B1 |
| 8.e.2 |  | 648 | B1 |
| 8.e.3 |  | 648 | B1 |
| 8.e.4 |  | 648 | B1 |
| 8f1 | 244 days post | 648 | B1 |
| 8f2 |  | NT |  |
| 8f3 |  | 648 | B1 |
| 8f4 |  | 648 | B1 |
| 8g1 | 330 days post | 131 | B2 |
| 8g2 |  | 131 | B2 |
| 8g3 |  | 38 | D |
| 8g4 |  | 131 | B2 |
| 9.1a | 28 days pre | NT |  |
| 9.1b |  | NT |  |
| 9.1c |  | NT |  |
| 9.1d |  | NT |  |
| 9.1e |  | NT |  |
| 9.1f |  | NT |  |
| 9.2a | 30 days pre | NT |  |
| 9.2b |  | 69 | D |
| 9.2c |  | 69 | D |
| 9.2d |  | 69 | D |
| 9.2e |  | 69 | D |
| 9.2f |  | 69 | D |
| 9.3a | 7 days pre | 3727 | NG |
| 9.3b |  | 3727 | NG |
| 9.3c |  | 3727 | NG |
| 9.3d |  | 3727 | NG |
| 9.3e |  | 3727 | NG |
| 9.3f |  | 3727 | NG |
| 9a1 | 5 days post | 4 | A |
| 9a2 |  | 200 | B1 |
| 9a3 |  | 4 | A |
| 9a4 |  | 4 | A |
| 9b1 | 14 days post | 2617 | F |
| 9b2 |  | 2617 | F |
| 9c1 | 35 days post | 2617 | F |
| 9c2 |  | 2617 | F |
| 9c3 |  | 2617 | F |
| 9c4 |  | 2617 | F |
| 9d1 | 91 days post | 2617 | F |
| 9d2 |  | NT | NG |
| 9d3 |  | 2617 | F |
| 9d4 |  | 2617 | F |
| 9.e.1 | 152 days post | 2617 | F |
| 9.e.2 |  | 2617 | F |
| 9.e.3 |  | 2617 | F |
| 9.e.4 |  | 2617 | F |
| 9f1 | 210 days post | 2617 | F |
| 9f2 |  | 2617 | F |
| 9f3 |  | 2617 | F |
| 9f4 |  | 2617 | F |
| 10.1a | 61 days pre | 354 | F |
| 10.1b |  | 354 | F |
| 10.1c |  | 354 | F |
| 10.1d |  | 354 | F |
| 10.1e |  | 354 | F |
| 10.1f |  | 354 | F |
| 10.2a | 30 days pre | 354 | F |
| 10.2b |  | 354 | F |
| 10.2c |  | 354 | F |
| 10.2d |  | 354 | F |
| 10.2e |  | 354 | F |
| 10.2f |  | 354 | F |
| 10.3a | 7 days pre | 642 | B1 |
| 10.3b |  | 642 | B1 |
| 10.3c |  | 642 | B1 |
| 10.3d |  | 642 | B1 |
| 10.3e |  | 642 | B1 |
| 10.3f |  | 642 | B1 |
| 10a1 | 5 days post | 200 | B1 |
| 10a2 |  | 34 | A |
| 10b1 | 14 days post | 200 | B1 |
| 10B2 |  | 200 | B1 |
| 10C1 | 35 days post | NT |  |
| 10E.1 | 152 days post | NT |  |
| 10E.2 |  | NT |  |
| 12.1a | 14 days pre | 1314 | A |
| 12.1b |  | 1314 | A |
| 12.1c |  | 10 | A |
| 12.1d |  | 10 | A |
| 12.1e |  | 1314 | A |
| 12.1f |  | 10 | A |
| 12.2a | 7 days pre | 10 | A |
| 12.2b |  | 10 | A |
| 12.2c |  | 752 | A |
| 12.2d |  | 10 | A |
| 12.2e |  | 770 | NG |
| 12.2f |  | 10 | A |
| 12a1 | 3 days post | 43 | A |
| 12a2 |  | 43 | A |
| 12a3 |  | 43 | A |
| 12a4 |  | 43 | A |
| 12b1 | 21 days post | 43 | A |
| 12b2 |  | 43 | A |
| 12b3 |  | 43 | A |
| 12b4 |  | 43 | A |
| 12c1 | 29 days post | 43 | A |
| 12c2 |  | 43 | A |
| 12c3 |  | 43 | A |
| 12c4 |  | 43 | A |
| 12d1 | 56 days post India | 43 | A |
| 12d2 |  | 43 | A |
| 12d3 |  | 43 | A |
| 12d4 |  | 43 | A |
| 12.e.1 | 115 days post India | 43 | A |
| 12.e.2 |  | 43 | A |
| 12.e.3 |  | 43 | A |
| 12.e.4 |  | 43 | A |
| 12fs | 213 days post India | 1276 | F |
| 12g1 | 305 days post India | 189 | A |
| 12g2 |  | 189 | A |
| 12g3 |  | 189 | A |
| 12g4 |  | 189 | A |
| 12h1 | 28 days post India | 5919 | NG |
| 12h2 |  | 5919 | NG |
| 12h3 |  | 216 | A |
| 12h4 |  | 5919 | NG |
| 15.1a | 28 days pre | 210 | NG |
| 15.1b |  | 210 | NG |
| 15.1c |  | 10 | A |
| 15.1d |  | 10 | A |
| 15.1e |  | 210 | NG |
| 15.1f |  | 210 | NG |
| 15.2a | 14 days pre | NT |  |
| 15.2b |  | NT |  |
| 15.2c |  | 10 | A |
| 15.2d |  | 10 | A |
| 15.2e |  | 210 | NG |
| 15.2f |  | 210 | NG |
| 15a1 | 1 day post | 167 | A |
| 15a2 |  | 167 | A |
| 15a3 |  | 167 | A |
| 15a4 |  | 167 | A |
| 15b | 122 days post | 52 | A |
| 15c1 | 150 days post | 10 | A |
| 15c2 |  | 10 | A |
| 15c3 |  | 10 | A |
| 15c4 |  | 10 | A |
| 16.1a | 42 days | 349 | D |
| 16.1b |  | 349 | D |
| 16.1c |  | 349 | D |
| 16.1d |  | 349 | D |
| 16.1e |  | 349 | D |
| 16.1f |  | 349 | D |
| 16.2a | 14 days | 349 | D |
| 16.2b |  | 349 | D |
| 16.2c |  | 349 | D |
| 16.2d |  | 349 | D |
| 16.2e |  | 349 | D |
| 16.2f |  | 349 | D |
| 16a1 | 7 days post | 38 | D |
| 16a2 |  | 38 | D |
| 16a3 |  | 38 | D |
| 16a4 |  | 38 | D |
| 16b1 | 40 days post | 227 | A |
| 16b2 |  | 131 | B2 |
| 16b3 |  | 38 | D |
| 16b4 |  | 227 | A |
| 16c1 | 91 days post | 131 | B2 |
| 16c2 |  | 131 | B2 |
| 16c3 |  | 227 | A |
| 16c4 |  | 131 | B2 |
| 16d1 | 121 days post | 131 | B2 |
| 16d2 |  | 131 | B2 |
| 16d3 |  | 131 | B2 |
| 16d4 |  | 131 | B2 |
| 16 e1 | 179 days post | 131 | B2 |
| 16 e2 |  | 131 | B2 |
| 16 e3 |  | 131 | B2 |
| 16 e4 |  | 131 | B2 |
| 17.1a | 30 days pre | NT |  |
| 17.1b |  | NT |  |
| 17.1c |  | NT |  |
| 17.1d |  | NT |  |
| 17.1e |  | NT |  |
| 17.1f |  | NT |  |
| 17.2a | 3 days pre | 131 | B2 |
| 17.2b |  | 131 | B2 |
| 17.2c |  | 131 | B2 |
| 17.2d |  | 131 | B2 |
| 17.2e |  | 131 | B2 |
| 17.2f |  | 131 | B2 |
| 17a1 | 21 days post | 450 | NG |
| 17a2 |  | 450 | NG |
| 17a3 |  | 450 | NG |
| 17a4 |  | 450 | NG |
| 17b1 | 42 days post | 43 | A |
| 17b2 |  | 43 | A |
| 17b3 |  | 43 | A |
| 17b4 |  | NT |  |
| 17c1 | 84 days post | 131 | B2 |
| 17c2 |  | 131 | B2 |
| 17c3 |  | 131 | B2 |
| 17c4 |  | 131 | B2 |
| 18.1a | 54 days pre | NS |  |
| 18.1b |  | NS |  |
| 18.1c |  | NS |  |
| 18.1d |  | NS |  |
| 18.1e |  | NS |  |
| 18.1f |  | NS |  |
| 18.2a | 36 days pre | NS |  |
| 18.2b |  | NS |  |
| 18.2c |  | NS |  |
| 18.2d |  | NS |  |
| 18.2e |  | NS |  |
| 18.2f |  | NS |  |
| 18a1 | 11 days post | NS |  |
| 18a2 |  | NS |  |
| 18a3 |  | NS |  |
| 18a4 |  | NS |  |
| 18b | 27 days post | NS |  |
| 19.1a | 42 days pre | 10 | A |
| 19.1b |  | 10 | A |
| 19.1c |  | 10 | A |
| 19.1d |  | 10 | A |
| 19.1e |  | 10 | A |
| 19.1f |  | 10 | A |
| 19.2a | 30 days pre | 10 | A |
| 19.2b |  | 10 | A |
| 19.2c |  | 10 | A |
| 19.2d |  | 10 | A |
| 19.2e |  | 10 | A |
| 19.2f |  | 10 | A |
| 19a1 | 14 days post | 43 | A |
| 19a2 |  | 43 | A |
| 19a3 |  | 43 | A |
| 19a4 |  | 43 | A |
| 20.1a | 10 days pre | 10 | A |
| 20.1b |  | 10 | A |
| 20.1c |  | 10 | A |
| 20.1d |  | 10 | A |
| 20.1e |  | 452 | NG |
| 20.1f |  | NT |  |
| 20.2a | 4 days pre | 10 | A |
| 20.2b |  | 10 | A |
| 20.2c |  | 10 | A |
| 20.2d |  | 10 | A |
| 20.2e |  | 10 | A |
| 20.2f |  | 10 | A |
| 20a1 | 7 days post | NT |  |
| 20a2 |  | NT |  |
| 20a3 |  | 162 | NG |
| 20a4 |  | 162 | NG |
| 20b1 | 42 days post | 162 | NG |
| 20b2 |  | 162 | NG |
| 20b3 |  | 162 | NG |
| 20b4 |  | 162 | NG |
| 20c1 | 56 days post | 131 | B2 |
| 20c2 |  | 226 | NG |
| 20c3 |  | 131 | B2 |
| 20c4 |  | 226 | NG |
| 20.e.3 | 91 days post | 405 | NG |
| 20.e.4 |  | 405 | NG |
| 20f1 | 120 days post | 10 | A |
| 20g1 | 183 days post | 10 | A |
| 20g2 |  | 10 | A |
| 20g3 |  | 10 | A |
| 20g4 |  | 10 | A |
| 21.1b | 16 days | NS |  |
| 21.1c |  | NS |  |
| 21.1d |  | NS |  |
| 21.1e |  | NS |  |
| 21.1f |  | NS |  |
| 21a1 | 15 days | NS |  |
| 21a2 |  | NS |  |
| 21a3 |  | NS |  |
| 21a4 |  | NS |  |
| 21b1 | 36 days | NS |  |
| 21b2 |  | NS |  |
| 21b3 |  | NS |  |
| 21b4 |  | NS |  |
| 21c1 | 96 days | NS |  |
| 21c2 |  | NS |  |
| 21c3 |  | NS |  |
| 21c4 |  | NS |  |
| 22.1a | 52 days | 2619 | B2 |
| 22.1b |  | 2619 | B2 |
| 22.1c |  | 2619 | B2 |
| 22.1d |  | 2619 | B2 |
| 22.1e |  | 2619 | B2 |
| 22.1f |  | 2619 | B2 |
| 22.2a | 11 days | 2619 | B2 |
| 22.2b |  | 2619 | B2 |
| 22.2c |  | 2619 | B2 |
| 22.2d |  | 2619 | B2 |
| 22.2e |  | 2619 | B2 |
| 22.2f |  | 2619 | B2 |
| 22a1 | 6 | 226 | A |
| 22bs | 14 | NS |  |
| 22c1 | 36 | 607 | A |
| 22c2 |  | 2967 | A |
| 22c3 |  | NT |  |
| 22c4 |  | 7174 | NG |
| NT: no sequence type based on Warwick MLST scheme; NG: no phylogenetic group assigned to the Warwick MLST; NS: not sequenced. Red and green shading represent non-CTX-M and CTX-M-producing isolates, respectively. | | | |
